# Heart Failure Awareness Day: A Tribute to the Genius Carlos Chagas

**DOI:** 10.5935/abc.20190137

**Published:** 2019-07

**Authors:** Evandro Tinoco Mesquita, Aurea Lucia Alves de Azevedo Grippa de Souza, Salvador Rassi

**Affiliations:** 1 Universidade Federal Fluminense, Niterói, RJ - Brazil; 2 Hospital Pró-Cardíaco, Rio de Janeiro, RJ - Brazil; 3 Departamento de Insuficiência Cardíaca da Sociedade Brasileira de Cardiologia (DEIC/SBC) - Diretoria Científica, Rio de Janeiro, RJ - Brazil; 4 Universidade Federal de Goiás, Goiânia, GO - Brazil; 5 Departamento de Insuficiência Cardíaca da Sociedade Brasileira de Cardiologia (DEIC/SBC) - Presidência, Rio de Janeiro, RJ - Brazil

**Keywords:** Heart Failure/epidemiology, Heart Failure /prevention and control, Risk Factors, Chagas Cardiomyopathy/physiopathology, Chagas Disease/epidemiology

The purpose of the Brazilian Society of Cardiology (SBC) is to expand and disseminate knowledge on Cardiovascular Science and to make each cardiologist aware of the activities intended to promote cardiovascular health in Brazilian society. Since its foundation, SBC has been stimulating research and dissemination, among civil society, of epidemiological and preventive matters related to the treatment of cardiovascular diseases, including heart failure (HF). HF has been identified as a cardiovascular epidemic throughout the world and involves important aspects on morbidity, mortality and healthcare costs, being little recognized among individuals, members of our society, healthcare managers and policy makers. The increasing prevalence of HF is due to population aging and the growth of risk factors, such as obesity, systemic arterial hypertension and diabetes mellitus, as well as increased survival of patients with heart diseases, such as congenital heart disease and ischemic heart disease. Worldwide, about 26 million adults have HF and forecasts indicate that the prevalence tends to increase by 25% by 2030.^1^

Within the scenario, it should be underscored that HF with preserved ejection fraction (HFPEF) needs greater recognition and prevention strategies by general practitioners, geriatricians and cardiologists.^2^ The syndrome presents asymptomatic stages and a symptomatic form, and preventive and therapeutic measures are able to reduce the progression of the disease and morbimortality. Outbreaks of acute HF and decompensation promote recurrence in emergency rooms and hospital admissions that promote worsening of the condition, where the outcome can be abrupt due to pump failure or sudden death.^3^ In this decade, initiatives in different countries, led by cardiology associations and societies, have promoted activities to warn the population and health professionals regarding early detection, complementary tests and access to medications and treatment based on scientific evidence.

**Figure 1:**
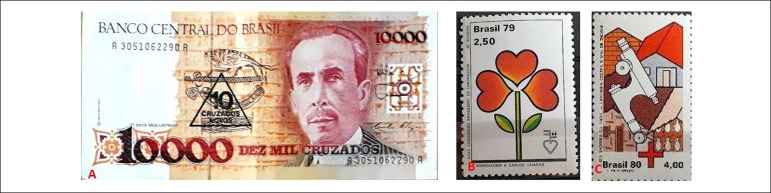
A) The ten thousand Brazilian cruzados note in honor of Carlos Chagas – “The notable physician in his laboratory” – launched in 1988. B) Stamp commemorating the 35th Brazilian Conference on Cardiology in honor of Carlos Chagas, 1979. C) Commemorative Stamp of the National Health Day – Chagas’ Disease, 1980.

Under the leadership of the Department of Heart Failure of the Brazilian Society of Cardiology (DEIC/SBC), Brazil is engaged in promoting activities related to this topic, by creating the awareness day, to be celebrated each year on July 9^th^, the data of birth of genius and pioneer cardiovascular translational scientist Carlos Justiniano Ribeiro das Chagas. This year, two dates are very remarkable: 140 years of the birth of professor and researcher Carlos Chagas and 110 years of the discovery of Chagas’ disease. A unique fact in the history of Medicine, in which a single researcher describes the vector, the etiological agent, identifies the hosts and attempts to identify the clinical forms of the disease.^4^ The theme of Chagas’ disease, in our country, remains contemporary, not only in the search for new treatments,^5^ vaccines and new forms of contamination, such as the oral route described in the north, northeast and south regions, involving sugarcane and açaí, reported this year in the Brazilian Archives of Cardiology,^6^ but also in the current outbreak of acute Chagas’ disease in Pernambuco, still in the process of clarification. Tribute has been paid to scientist Carlos Chagas in Brazil in several ways (Figure 1), but we have identified that his contribution to cardiology and HF was definitive and important, representing his remarkable name for this noble cause in our country.

With continental dimensions, our country has a great diversity of lifestyle, environmental, socioeconomic and cultural risk factors, as well as the composition of its health system (access, organization of the healthcare network, financing, availability of technological resources and professionals). IC clinics that can offer multiprofessional care have been shown to reduce admissions and improve quality of life. Cardiac rehabilitation has proven effectiveness, but it is not yet available, causing the low inclusion of people in these programs. Different factors may explain the variability of the number of hospital admissions, early retirement for HF and use of resources - heart transplantation, surgery, cardiac catheterization, pacemaker implantation, defibrillators and healthcare costs. According to Araujo et al.,^7^ the direct cost of treatment is represented mainly by hospitalization and costs with medication. However, indirect costs represent economic impacts similar to direct costs. A recent study estimates that about BRL 22.1 billion/US$ 6.8 billion were spent on the treatment of HF in our country in 2015 alone,^8^ and could represent 68% of hospitalization costs, as seen in the USA and Europe, due to costly diagnostic techniques, high cost of medications, interventions and devices. Therefore, reducing hospital admissions is critical for reducing costs, in addition to the physical and psychological limitations that can be worsened in these patients, particularly depressive symptoms and anxiety. The increasing prevalence of hospital admissions, estimated in some centers at up to 25%, and high hospital morbidity and mortality rates, especially in the presence of comorbidities, such as renal failure, have been documented in different studies.^9,10^ Private sector data are beginning to be described and published as those present at the Observatory of the National Association of Private Hospitals (ANAHP) on patients admitted due to HF in a group of hospitals with accreditation and protocols to serve this population. Data from the 2019 Observatory reveal,^11^ in 2017 and 2018, respectively, a median of stay of 7.56 and 6.72 (SD = 3.72), inpatient mortality of 7.49% and 5.26%, rate of use of angiotensin converting enzyme (ACE) inhibitors or angiotensin receptor blockers (ARA) on discharge of 89.43% and 88.41% (SD = 17.40%) and rate of use of betablockers at discharge of eligible patients of 93.29% and 94.29% (SD = 10.09%).

Being aware of this variability in healthcare and outcomes of HF is important for us to help improve the quality of care in our country. Through the BREATHE registry, DEIC has studied patients from different regions of the country and verified the high in-hospital mortality among those admitted with acute HF associated with low rate of prescription of evidence-based medicines in Brazil.^12^ Fonseca et al.^13^ have found that through the demographic changes observed over the years in continental Portugal and the clinical practices currently employed, in about 40 years, the country will have half a million patients undergoing treatment for HF, highlighting the extreme need for raising people’s awareness, improving reference levels and healthcare so that the burden of the syndrome in the country may have its representativity diminished. They also stress the importance of optimizing healthcare strategies, organizing essential healthcare services, promoting adjustments while respecting regional characteristics, by avoiding a single model of work, reiterating the need for organized discussions at all levels of healthcare to the population.^14^ Corroborating this need for adjusting and regionalizing healthcare, Kaufman et al.^15^ argue that, considering the last 12 years of HF in Brazil, the Southeast region presented the highest number of hospital admissions, accounting for 41.4% of hospital admissions according to DATASUS data. In the characterization of this population in South America, Brazil contributes with most of the studies, accounting for 64% of the production of published data, being the only one to present its incidence in a population study.^16^

DEIC/SBC has continuously used its Acute and Chronic Heart Failure Guideline published in 2018 to ensure the best scientific evidence available on diagnosis and treatment.^3^ Regarding the role of diagnosis and beginning of treatment, many patients are still diagnosed during their first admission for acute HF. This demonstrates the need for greater investment in the continuing education of general practitioners, clinicians and cardiologists working at family clinics, basic health units and practices, and in the multidisciplinary and organized healthcare, in order to coordinate the line of care, palliative care protocols, in short, medical care based on HF teams. In the experience of Germany, 63.2% of the new cases were identified in a doctor’s office and 94% of diagnosed patients received their first prescription from the general practitioner. Cases of HF in hospitalized patients, where previous diagnosis was given by a non-specialist in 70.7%, reaffirm the strategy of investing in mechanisms of recognition of the disease in this group of professionals.^17^ In our country, we do not have a healthcare process based on the full care of patients in a standardized way, which leads to delayed diagnosis in many patients, without the recognition of signs and symptoms by individuals and caregivers. This said, the process of education, recognition of the main symptoms and treatment are imperative needs, and initiatives that can promote knowledge about HF are fundamental for improving the quality of care. Besides those, access to complementary tests that demonstrate the objective presence of HF, such as the biomarkers brain natriuretic peptide (BNP) and N-terminal prohormone of brain natriuretic peptide (NT pro-BNP), and imaging scans for the correct handling of these patients.

It is necessary to emphasize the importance of obtaining population registries to draw up a global picture of HF. China, Malaysia and South Africa, involved in the International Congestive Heart Failure (Inter-CHF) study, show that although socioeconomic similarities are distinct in etiology, comorbidities, sociodemographic characteristics and outcomes,^18^ some comorbidities, such as diabetes mellitus, atrial fibrillation and chronic kidney disease, have been shown to be independent factors associated with hospital admissions and mortality in some samples. Hospital admissions and visits to the emergency room were also shown to be associated with outcomes in several studies.^19^ Despite these well-established comorbidities correlated to outcomes, we found a group whose role is not yet properly characterized, such as sleep apnea, iron deficiency, sarcopenia and chronic obstructive pulmonary disease. Further studies regarding the contribution of these comorbidities in mortality still need to be developed.^20^ Learning about disparate socioeconomic conditions that modify the incidence of HF in different regions in the same country can also be derived from population registries. Even in health systems such as that of the UK, it can be seen that social inequalities interfere with accessibility and lead to a consequent increase in cases of the syndrome.^21^ As for mortality, using hospital records and death certificates for the mapping out HF, we have observed that, so it can be better characterized, we need to validate a standardized registration methodology using more accurate statistical analyses. The inconsistencies between hospital records and reports of cause of death are presented as a window of opportunity for the implementation of studies to improve quantification in all regions of Brazil.^22^ Along with the Brazilian Heart Failure Network (REBRIC), we intend to mobilize all the instances involved in healthcare, disseminating ideas and sharing knowledge to improve healthcare.^23^

HF specialists and cardiologists have the important challenge, at the present time, of organizing the health assistance process in the coordination of lines and resources, aiming at expanding the connection with general practitioners, emergency surgeons, geriatricians and hospitalists, promoting the improvement of clinical outcomes and the patient’s journey. The project that we call the HF Map will be one of the priorities of the Department of Heart Failure of the Brazilian Society of Cardiology for 2020-2021, following the consolidated model of comprehensive epistemological assessment of HF figures in our country, tested by the European Society of Cardiology (ESC) and by the European Society of Heart Failure (ESHF/ESC).^24^ This will be done through a digital platform that will collect data from different regions in order to quantify and make available the figures of this epidemic in our country. This platform will be implemented in 2020 and will include national data from the public and private sectors, from regulated organizations and scientific publications, which will be evaluated by data scientists, cardiovascular epidemiologists and clinical and population health researchers. Within this plan, the 1^st^ Registry of Cardiomyopathies and Myocarditis in Children and Adolescents will be carried out in collaboration with the Group of Studies in Cardiomyopathies (GEMIC/SBC), aiming at mapping out and describing this population to contribute with better proposals of continuing medical education, clinical diagnostic criteria and creation of specialized centers of treatment and care.

**Figure 2:**
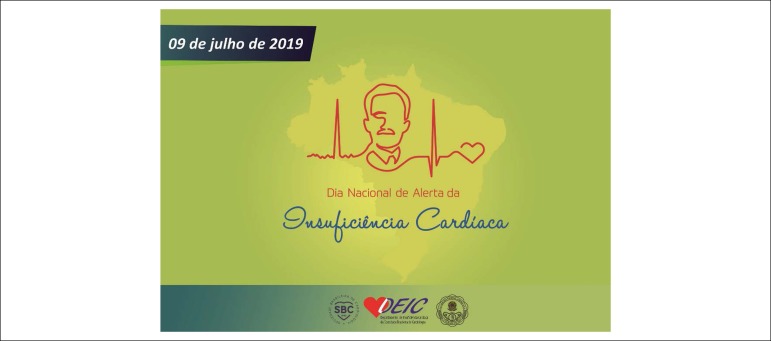
This is the logo of the heart failure awareness day campaign promoted by DEIC/SBC, supported by the National Academy of Medicine.

In short, the construction of a day of awareness on HF (Figure 2), on Carlos Chagas’ date of birth, aims at raising the awareness for healthcare professionals, patients, relatives and society in general; and the decision makers in health, who can make decisions and take actions that can influence the evolution of this important cardiovascular epidemic in Brazil. This is one of DEIC’s commitments, which is in lined with the mission of SBC, and supported by the National Academy of Medicine.
